# Identification of a tumor microenvironment-related gene signature to improve the prediction of cervical cancer prognosis

**DOI:** 10.1186/s12935-021-01867-2

**Published:** 2021-03-25

**Authors:** Qian Chen, Bingqing Qiu, Xiaoyun Zeng, Lang Hu, Dongping Huang, Kaihua Chen, Xiaoqiang Qiu

**Affiliations:** 1 Department of Research, Guangxi Medical University Cancer Hospital, Nanning, Guangxi 530021 China; 2 Department of Nuclear Medicine, Guangxi Medical University Cancer Hospital, Nanning, Guangxi 530021 China; 3 Department of Epidemiology, School of Public Health, Guangxi Medical University, Nanning, Guangxi 530021 China; 4 Guangxi Medical University Cancer Hospital, Nanning, Guangxi 530021 China; 5 Department of Nutrition, School of Public Health, Guangxi Medical University, Nanning, Guangxi 530021 China; 6 Department of Radiation Oncology, Guangxi Medical University Cancer Hospital, Nanning, Guangxi 530021 China

**Keywords:** Cervical cancer, Tumor microenvironment, TCGA, Prognostic signature

## Abstract

**Background:**

Previous studies have found that the microenvironment of cervical cancer (CESC) affects the progression and treatment of this disease. Thus, we constructed a multigene model to assess the survival of patients with cervical cancer.

**Methods:**

We scored 307 CESC samples from The Cancer Genome Atlas (TCGA) and divided them into high and low matrix and immune scores using the ESTIMATE algorithm for differential gene analysis. Cervical cancer patients were randomly divided into a training group, testing group and combined group. The multigene signature prognostic model was constructed by Cox analyses. Multivariate Cox analysis was applied to evaluate the significance of the multigene signature for cervical cancer prognosis. Prognosis was assessed by Kaplan–Meier curves comparing the different groups, and the accuracy of the prognostic model was analyzed by receiver operating characteristic-area under the curve (ROC-AUC) analysis and calibration curve. The Tumor Immune Estimation Resource (TIMER) database was used to analyze the relationship between the multigene signature and immune cell infiltration.

**Results:**

We obtained 420 differentially expressed genes in the tumor microenvironment from 307 patients with cervical cancer. A three-gene signature (SLAMF1, CD27, SELL) model related to the tumor microenvironment was constructed to assess patient survival. Kaplan–Meier analysis showed that patients with high risk scores had a poor prognosis. The ROC-AUC value indicated that the model was an accurate predictor of cervical cancer prognosis. Multivariate cox analysis showed the three-gene signature to be an independent risk factor for the prognosis of cervical cancer. A nomogram combining the three-gene signature and clinical features was constructed, and calibration plots showed that the nomogram resulted in an accurate prognosis for patients. The three-gene signature was associated with T stage, M stage and degree of immune infiltration in patients with cervical cancer.

**Conclusions:**

This research suggests that the developed three-gene signature may be applied as a biomarker to predict the prognosis of and personalized therapy for CESC.

## Background

Despite expanding the screening scale of cervical cancer (CESC) and promoting the use of vaccines, there were 569,847 new cases of CESC and 311,365 deaths from CESC worldwide in 2018. The incidence and mortality rate of CESC were the fourth highest among all cancers in women, suggesting a serious societal burden [1]. High-risk human papillomavirus (HR-HPV) persistent infection is the main cause of cervical cancer development [2]. Cervical squamous cell carcinoma and cervical adenocarcinoma are the main types of cervical cancer. At present, the conventional treatment of cervical cancer includes radiotherapy, chemotherapy and surgery, but patients at advanced stages are prone to developing radiotherapy and chemotherapy resistance. Many patients with cervical cancer are already in the advanced stage at the time of diagnosis, which is often accompanied by high invasion rates, and the 3-year or 5-year mortality rate is between 52 and 79% [3–5]. Although some diagnostic and prognostic markers for cervical cancer have been discovered, the overall efficacy of these markers remains a challenge because of the heterogeneity of cervical cancer. Therefore, it is necessary to find prognostic markers for cervical cancer at the molecular level, which will offer new insight into the treatment and prognosis of cervical cancer.

Previous articles have revealed that the balance of the host tumor microenvironment (TME) plays a vital role in the process of cancers. The TME consists of fibroblasts, vascular endothelial cells, immune cells, cytokines, growth factors, hormones, extracellular matrix components, and so on [6]. The TME affects invasion, migration, proliferation, apoptosis, and response to drugs in CESC cells [7–11]. The Estimation of Stromal and Immune cells in MAlignant Tumor tissues using Expression data (ESTIMATE) algorithm uses gene expression data to analyze the proportion of immune cells and stromal cells in the TME [12]. The algorithm is based on single-sample gene set enrichment analysis (ssGSEA), and the immune score, matrix score and tumor purity score are obtained. TME scores have been reported to aid in the prognosis of glioblastoma and colorectal cancer patients [13, 14].

As the largest cancer gene database at present, The Cancer Genome Atlas (TCGA) database has rich sequencing and microarray data, including gene expression, miRNA expression, copy number variation, DNA methylation, SNP and standardized clinical data. Previous studies have found that multigene models can predict tumor prognosis base on TCGA. In breast cancer, Qi et al. found that a prognostic model of a four-gene signature predicted the survival of breast cancer patients [15]. A recent article involving papillary thyroid cancer showed that a prognostic model of an seven-gene signature predicted patient outcomes and was associated with stage and metastasis [16]. In addition, He et al. indicated a prognostic model of an eight-gene signature to be an independent risk factor for the prognosis of lung cancer, providing new ideas for accurate treatment of patients [17]. Multigene models are also important for individualized systemic treatment, prolonged survival and early detection of cervical cancer. A recent study showed that a 70-gene signature can be used as a predictor of the therapeutic effect of individualized systemic treatment of advanced-stage cervical cancer, which can help prolong the survival time of patients [18]. Xie et al. found that an 8-gene signature can predict the prognosis of patients with cervical cancer after radiotherapy [19]. Cai et al. reported that a glycolysis-related six-gene signature can be used as a prognostic marker to accurately predict the prognosis of cervical cancer patients [20]. Ma et al. found that a 4-gene signature in peripheral plasma is a new marker for the diagnosis of cervical cancer and has good diagnostic performance [21]. However, a multigene model associated with the tumor microenvironment (TME) has not yet been reported for assessing survival in cervical cancer.

In this study, we used the ESTIMATE algorithm to determine the immune and matrix scores of CESC samples to identify hub genes in the TME. We constructed a prognostic model of multigene signatures by performing a multivariate Cox regression analysis of prognosis-associated hub genes in the TME using the ‘‘survival’’ package in R software. According to our results, the three-gene signature (SLAMF1, CD27, SELL) may be applied as a biomarker to predict prognosis and to provide new insight for therapy.

## Methods

### Data preparation

We obtained mRNA expression data and patient clinical information from the TCGA database (https://cancergenome.nih.gov/). The clinical data included pathological tumor type, patient age, tumor stage, tumor grade, follow-up time and status. The exclusion criteria were set as follows: (1) histologic diagnosis is not CESC; (2) samples lack gene expression profile for analysis; and (3) clinical information is not available for analysis. Overall, a total of 307 CESC patients from the TCGA database were included in our study. The patients were randomly divided into a training group and a testing group at a ratio of 1:1.

### Screening of differentially expressed genes in the TME

The ratio of immune to stromal cells in the TME was calculated using the ESTIMATE algorithm, and the samples were separated into high- and low-score groups based on the median value for differential analysis. Gene expression values were analyzed using the limma package in R 3.5.3. The expression value of each gene was log2-converted for further study, and the differential genes with a log2-fold change (log2FC) > 1 and a false discovery rate (FDR) < 0.05 were considered significant.

### Biological functionality analyses

Gene Ontology (GO) functional annotation and Kyoto Encyclopedia of Genes and Genomes (KEGG) pathway enrichment analysis are commonly used to assess the functions of genes. We used the clusterProfiler package in R to perform GO and KEGG analyses on the differential genes in the microenvironment. The STRING 11.0 database was utilized to discover protein–protein interactions (PPIs), and the Molecular Complex Detection (MCODE) 1.5.1 plugin was used to find core genes [22]. The PPI network was visualized using Cytoscape 3.7.1 [23].

### Analysis of immune cell infiltration

The Tumor Immune Estimation Resource (TIMER) system can be found at https://cistrome.shinyapps.io/timer/. The TIMER database system was used to analyze the immune infiltration of the samples from the TCGA database, including 10,897 tissues and 32 cancer types [24]. We used the TIMER database to study the relationship between the risk score of three-gene signature and immune cell infiltration in CESC tissues.

### Statistical analysis

Construction of a multigene signature prognostic model by Cox model analysis was carried out using the ‘‘survival’’ package in R software. The gene signature can be predicted as follows: risk score = ∑βmRNAi × ExpmRNAi (where β is the coefficient and Exp is expression of the gene). Survival analysis was used to compare the relationship between different risk scores (high score vs low score) and prognosis. The accuracy of the prognostic evaluation of the risk score of the three-gene signature was evaluated based on the ROC analysis. The same formula was used to calculate the risk score in the testing group and the combined group (training group + testing group) to verify the accuracy of the model. Multivariate cox analysis of the multigene signature was performed to assess whether it is an independent prognostic risk factor. Nomogram analysis and calibration plot were conducted using the “rms” package. The association between the risk score and immune cell infiltration was analyzed by Spearman correlation. The data were analyzed using R software. Statistical significance was set as a P-value < 0.05.

## Results

### Matrix score and immune score of the CESC microenvironment

**Fig. 1:**
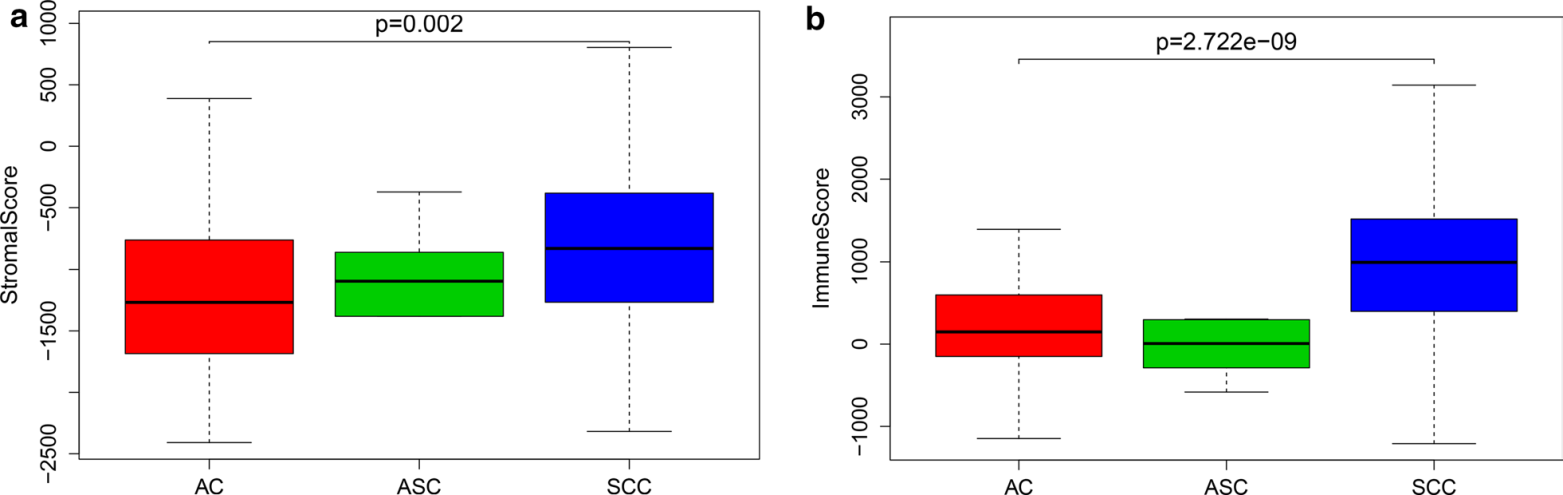
Stromal scores and immune scores are related to pathological types of cervical cancer. **a** Range of stromal scores for different cervical cancer pathologies (SCC > ASC > AC). **b** Range of immune scores for different cervical cancer pathologies (SCC > AC > ASC)

We downloaded the clinical information and gene expression matrices of 307 CESC patients from TCGA. The mean age of these patients was 48 years. A total of 254 patients were diagnosed with squamous cell carcinoma (SCC), 46 patients were diagnosed with adenocarcinoma (AC), and 6 patients were diagnosed with adenosquamous carcinoma (ASC). To study the ratio of stromal cells and immune cells in the TME, we used the ESTIMATE algorithm to analyze gene expression data. The matrix score ranged from − 2437.39 to 804.22, and the average value was -868.78; the immune score ranged from − 1209.74 to 3419.33, and the average value was 828.83. The results showed that SCC had the highest matrix score, followed by ASC, and AC had the lowest matrix score (Fig. 1a, P = 0.002). The order of the immune score from high to low was SCC > AC > ASC (Fig. 1b, P = 2.72E−09), suggesting that the pathological type of CESC affects the matrix score and the immune score.

### Expression of genes in different matrix and immune score groups

**Fig. 2:**
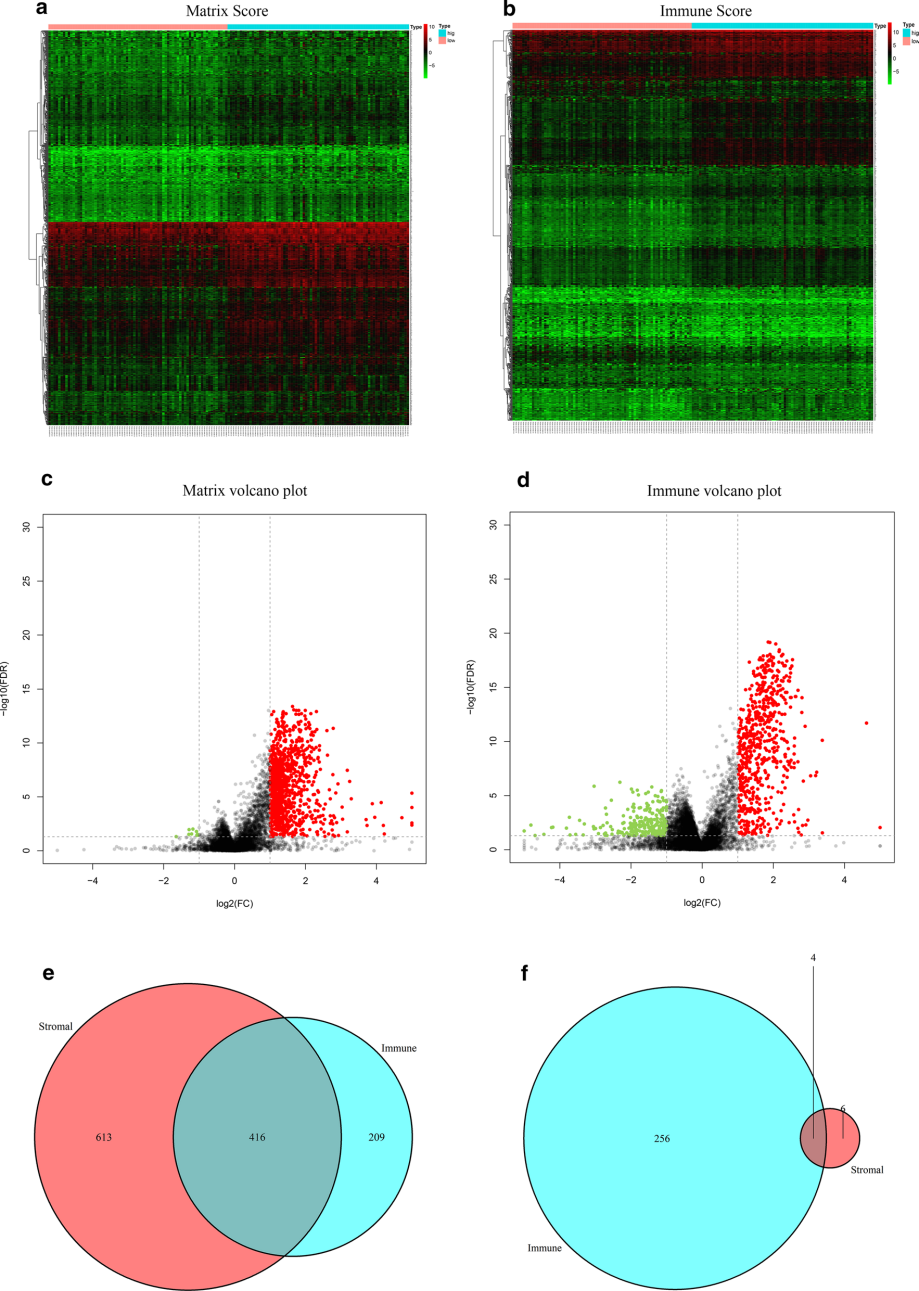
Differentially expressed genes in the cervical cancer microenvironment. Heatmap and volcano plots show the differentially expressed genes in the cervical cancer microenvironment. Red represents a high expression of the gene in the sample, and green represents a low expression of the gene in the sample. (Log2-fold change > 1, false discovery rate < 0.05). **a** Heatmap of the differential genes with matrix scores (high score vs low score). **b** Heatmap of the differential genes with immune scores (high score vs low score). **c** Volcano plots of the differential genes with matrix scores (high score vs low score). **d** Volcano plots of the differential genes with immune scores (high score vs low score). **e** Venn diagram showing 416 upregulated genes in the cervical cancer microenvironment. **f** Venn diagram showing 4 downregulated genes in the cervical cancer microenvironment

We included 307 patients in the cohort to study the expression of genes in different stromal and immune score groups. Genes with a fold change > 2 and an FDR < 0.05 were included in the subsequent analysis. As a result, 1029 genes were highly expressed in the high matrix-score group, and 10 genes were expressed at low levels (Fig. 2a, c). There were 625 genes with high expression in the high immune-score group and 260 genes with low expression (Fig. 2b, d). The Venn diagram shows that a total of 416 genes were upregulated in the stromal/immune high-score group, and 4 genes were downregulated in the stromal/immune high-score group. These genes were considered to be differentially expressed genes in the microenvironment (Fig. 2e, f).

### GO, KEGG, and PPI analyses of differentially expressed genes

**Fig. 3:**
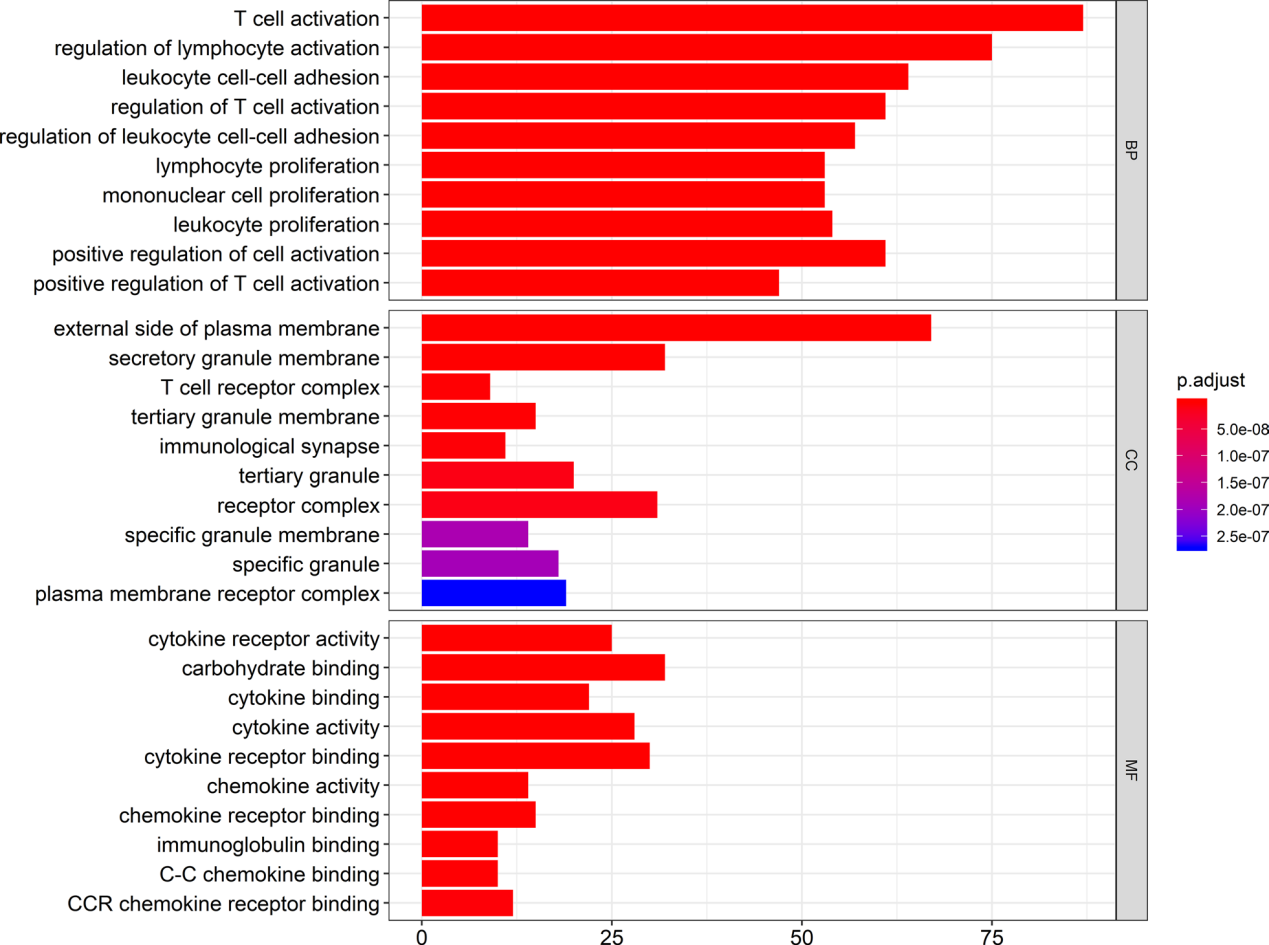
GO terms of the differentially expressed genes

**Fig. 4:**
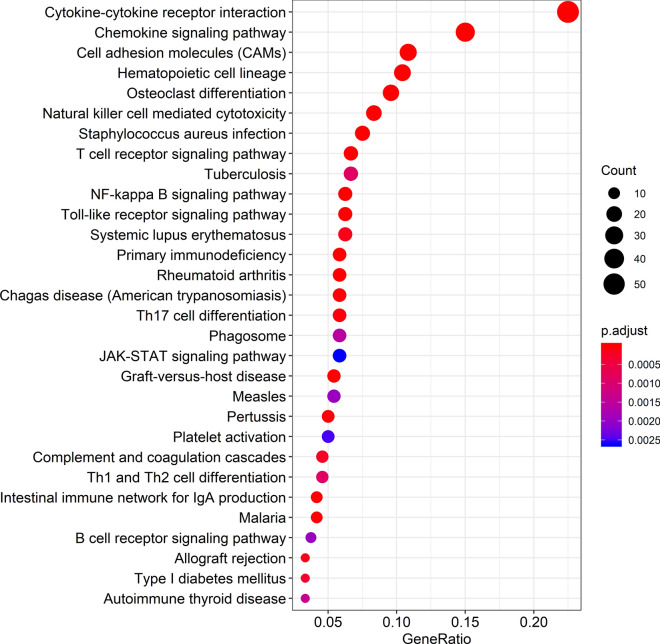
KEGG pathways of the differentially expressed genes

**Fig. 5:**
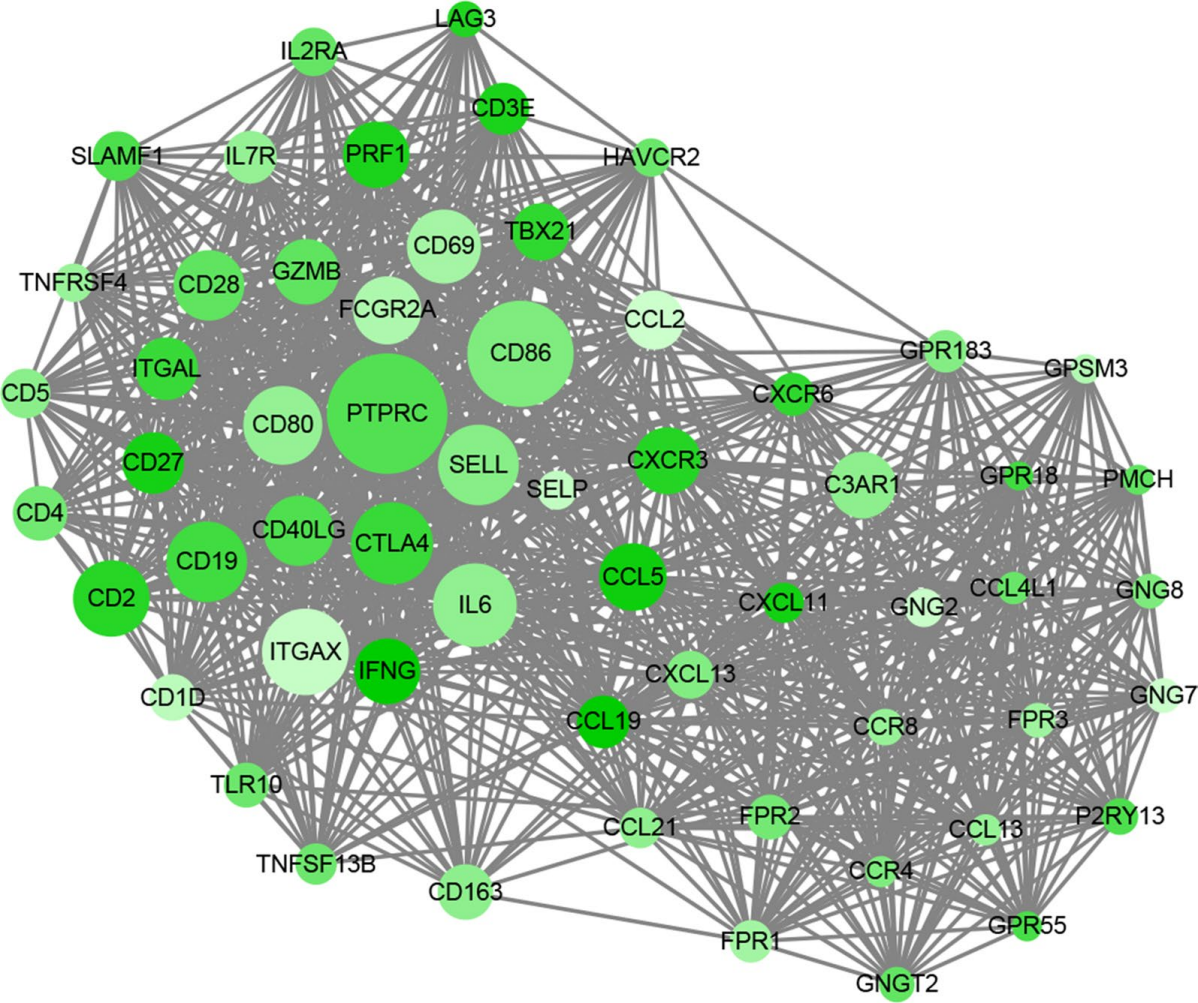
Protein–protein interaction network of hub proteins in the cervical cancer microenvironment. The MCODE plugin was used to screen modules of the PPI network using Cytoscape 3.7.1. The core module was found to consist of 59 nodes and 954 edges (MCODE score: 32.897)

To study the role of these genes in the CESC microenvironment, we used GO and KEGG analyses. The biological process (BP) category contained T cell activation and leukocyte cell–cell adhesion. T cell receptor complex and immunological synapse were included in the GO cellular component (CC) category. The molecular function (MF) category included cytokine receptor activity and CCR chemokine receptor binding (Table 1; Fig. 3). The results of the KEGG pathway enrichment are displayed in Table 2; the genes were mainly enriched in immune-related pathways, including the T cell receptor signaling pathway, intestinal immune network for IgA production and B cell receptor signaling pathway (Fig. 4). The PPI network analysis was based on the STRING database and included 392 nodes and 7228 edges. Then, we used the MCODE plugin in Cytoscape to find the core genes. A total of 59 genes were considered to be core genes and were used for subsequent analysis (Fig. 5).

**Table 1:** GO terms of enriched genes

No	GO ID	GO term	*P*-adjust		Count
Biological process					
1	GO:0042110	T cell activation	9.42E−56		87
2	GO:0051249	Regulation of lymphocyte activation	2.58E-41		75
3	GO:0007159	Leukocyte cell–cell adhesion	1.21E−40		64
4	GO:0050863	Regulation of T cell activation	2.71E−39		61
5	GO:1903037	Regulation of leukocyte cell–cell adhesion	6.44E−36		57
6	GO:0046651	Lymphocyte proliferation	4.99E−34		53
7	GO:0032943	Mononuclear cell proliferation	6.44E−34		53
8	GO:0070661	Leukocyte proliferation	1.25E−33		54
9	GO:0050867	Positive regulation of cell activation	1.52E−33		61
10	GO:0050870	Positive regulation of T cell activation	2.90E−33		47
Cellular component					
1	GO:0009897	External side of plasma membrane	5.39E−42		67
2	GO:0030667	Secretory granule membrane	2.69E−12		32
3	GO:0042101	T cell receptor complex	1.23E−09		9
4	GO:0070821	Tertiary granule membrane	1.42E−09		15
5	GO:0001772	Immunological synapse	2.68E−09		11
6	GO:0070820	Tertiary granule	9.55E−09		20
7	GO:0043235	Receptor complex	9.91E−09		31
8	GO:0035579	Specific granule membrane	1.83E−07		14
9	GO:0042581	Specific granule	1.90E−07		18
10	GO:0098802	Plasma membrane receptor complex	2.71E−07		19
Molecular function					
1	GO:0004896	Cytokine receptor activity	2.82E−18		25
2	GO:0030246	Carbohydrate binding	9.65E−13		32
3	GO:0019955	Cytokine binding	4.21E−12		22
4	GO:0005125	Cytokine activity	4.27E−12		28
5	GO:0005126	Cytokine receptor binding	5.07E−11		30
6	GO:0008009	Chemokine activity	9.80E−11		14
7	GO:0042379	Chemokine receptor binding	5.07E−10		15
8	GO:0019865	Immunoglobulin binding	9.96E−10		10
9	GO:0019957	C–C chemokine binding	1.49E−09		10
10	GO:0048020	CCR chemokine receptor binding	2.91E−09		12

**Table 2:** KEGG pathways of enriched genes

No	KEGG ID	KEGG term	*P*-adjust	Count
1	hsa04060	Cytokine–cytokine receptor interaction	8.57E−26	54
2	hsa04062	Chemokine signaling pathway	2.99E−17	36
3	hsa04514	Cell adhesion molecules (CAMs)	8.74E−12	26
4	hsa04640	Hematopoietic cell lineage	3.57E−15	25
5	hsa04380	Osteoclast differentiation	1.10E−10	23
6	hsa04650	Natural killer cell mediated cytotoxicity	4.65E−08	20
7	hsa05150	Staphylococcus aureus infection	3.54E−11	18
8	hsa04660	T cell receptor signaling pathway	1.04E−06	16
9	hsa05152	Tuberculosis	8.60E−04	16
10	hsa04064	NF-kappa B signaling pathway	5.23E−06	15
11	hsa04620	Toll-like receptor signaling pathway	8.06E−06	15
12	hsa05322	Systemic lupus erythematosus	1.18E−04	15
13	hsa05340	Primary immunodeficiency	4.36E−11	14
14	hsa05323	Rheumatoid arthritis	8.06E−06	14
15	hsa05142	Chagas disease	3.04E−05	14
16	hsa04659	Th17 cell differentiation	4.29E−05	14
17	hsa04145	Phagosome	1.56E−03	14
18	hsa04630	JAK-STAT signaling pathway	2.62E−03	14
19	hsa05332	Graft-versus-host disease	2.84E−09	13
20	hsa05162	Measles	1.95E−03	13
21	hsa05133	Pertussis	3.05E−05	12
22	hsa04611	Platelet activation	2.50E−03	12
23	hsa04610	Complement and coagulation cascades	2.22E−04	11
24	hsa04658	Th1 and Th2 cell differentiation	8.49E−04	11
24	hsa04672	Intestinal immune network for IgA production	2.12E−05	10
26	hsa05144	Malaria	2.12E−05	10
27	hsa04662	B cell receptor signaling pathway	1.95E−03	9
28	hsa05330	Allograft rejection	1.40E−04	8
29	hsa04940	Type I diabetes mellitus	3.30E−04	8
30	hsa05320	Autoimmune thyroid disease	1.36E−03	8

### Identification of the three-gene signature characteristics

**Fig. 6:**
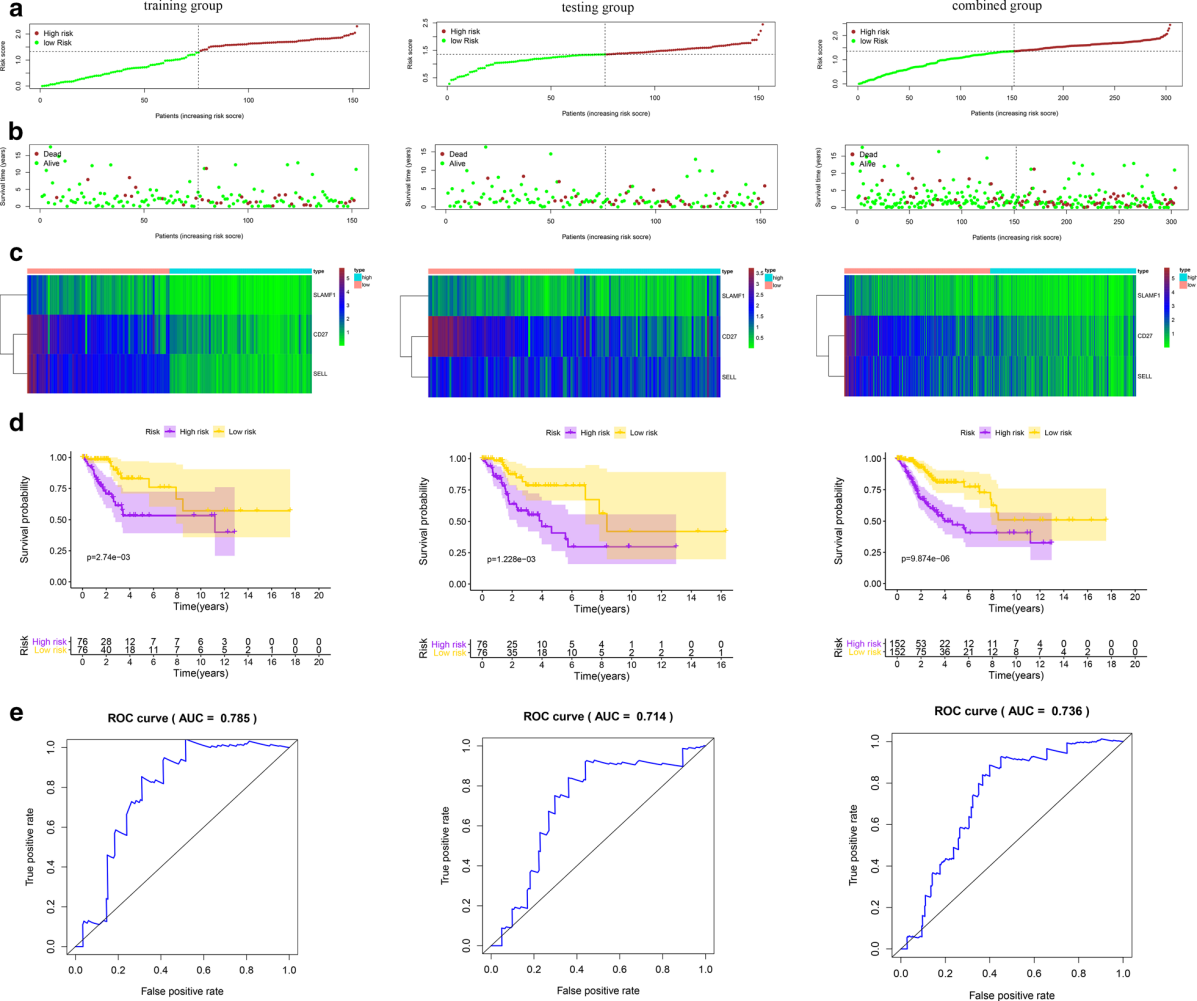
Risk score of the prognostic signature comprising the three-gene signature for overall survival (OS) in the three groups. **a** Distribution of patients with different risk scores in the training, testing and combined groups. **b** OS status of patients with different risk scores in the training, testing and combined groups. **c** Heatmap of the prognostic signature scores in the training, testing and combined groups. **d** Kaplan–Meier (K–M) analysis of patients in the high- and low-risk groups in the training, testing and combined groups. **e** Verification of the prognostic value of the three-gene signature by ROC analysis in the training, testing and combined groups

**Fig. 7:**
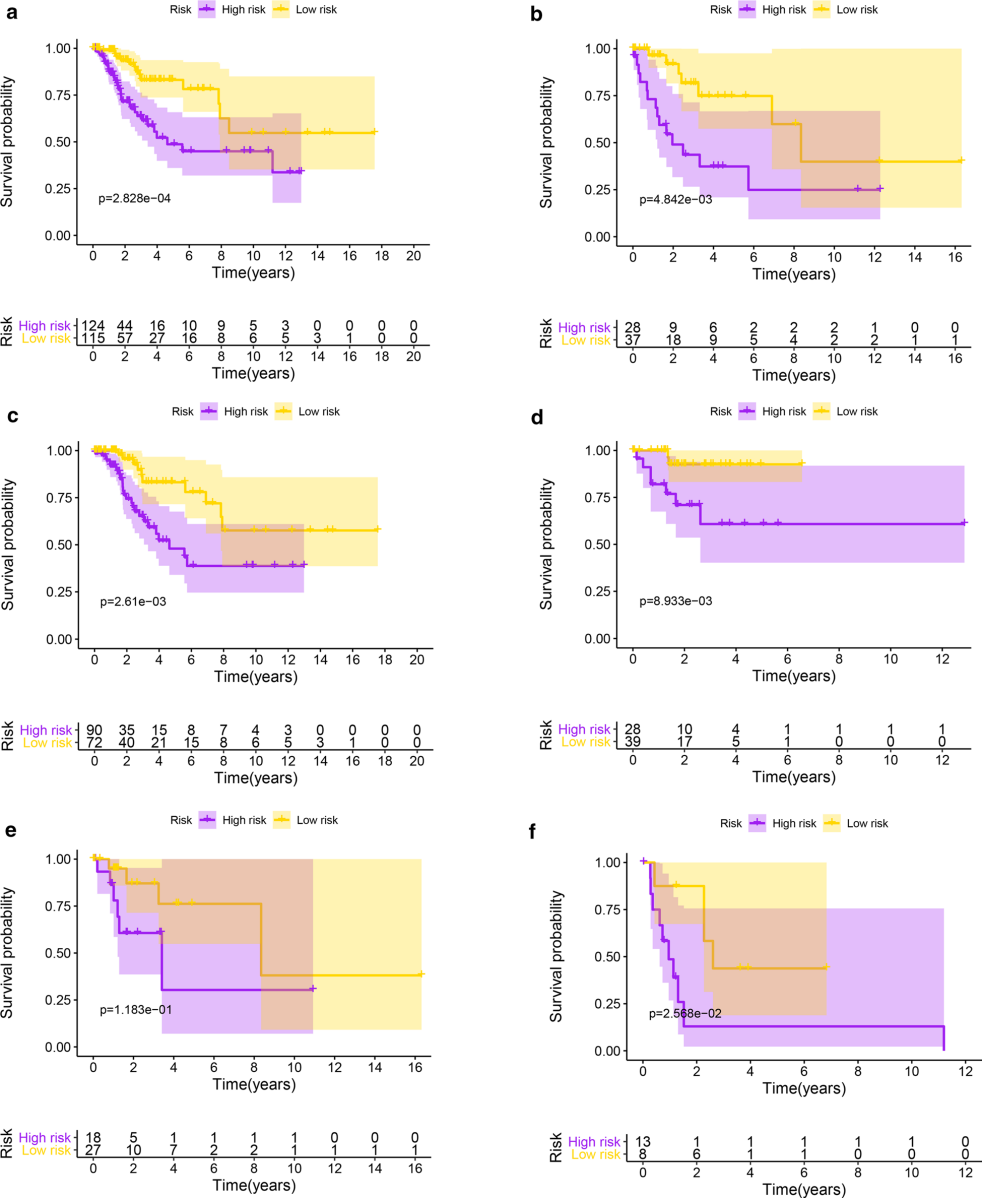
Subgroup analyses of OS for cervical cancer patients. Subgroup analysis of the three-gene signature for (**a**) age < 60; **b** age ≥ 60; **c** stage I; **d** stage II; **e** stage III; and **f** stage IV

In the training group, we performed univariate Cox regression analysis to identify the core genes associated with overall survival, which showed that six core genes were associated with overall survival (P < 0.05). Then, we constructed a prognostic model of multigene signatures by performing a multivariate Cox regression analysis of prognosis-associated core genes using the ‘‘survival’’ package in R software. The results show that the three-gene signature (SLAMF1, CD27, SELL) can be predicted as follows: Risk Score = [SLAMF1 × (0.7380)] + [CD27 × (− 0.1894)] + [SELL × (− 0.1008)] (Table 3). The patient’s risk score was evaluate based on expression of the three genes, and the patients were divided into groups of high and low scores according to the median value (Fig. 6). The results showed that the overall survival time of the high-risk score patients was shorter than that of the low-risk score patients; the 5-year survival rate of the high-risk score patients was 55.3%, and that of the low-risk score patients was 76.1% (Fig. 6d). ROC analysis showed that the three-gene signature can accurately predict the survival of patients with cervical cancer in the training group (AUC = 0.785, Fig. 6e). In addition, we performed verification analysis of the three-gene signature in the testing group and combined group. Survival analysis showed that the 5-year survival rate in the high-risk group was lower than that in the low-risk group (Fig. 6d). The AUCs for the testing group and combined group were 0.714 and 0.736, respectively (Fig. 6e). Prognostic analysis also showed that the three-gene signature can predict prognosis among patients at different ages and stages (Fig. 7a–f).

**Table 3:** A three-gene signature in cervical cancer microenvironment

ID	Coef	HR	HR.95L	HR.95H	P value
SLAMF1	0.738	2.092	0.835	5.242	1.154E−01
CD27	− 0.189	0.827	0.698	0.981	2.955E−02
SELL	− 0.101	0.904	0.798	1.024	1.138E−01

### The three-gene signature is an independent risk factor of prognosis

**Fig. 8:**
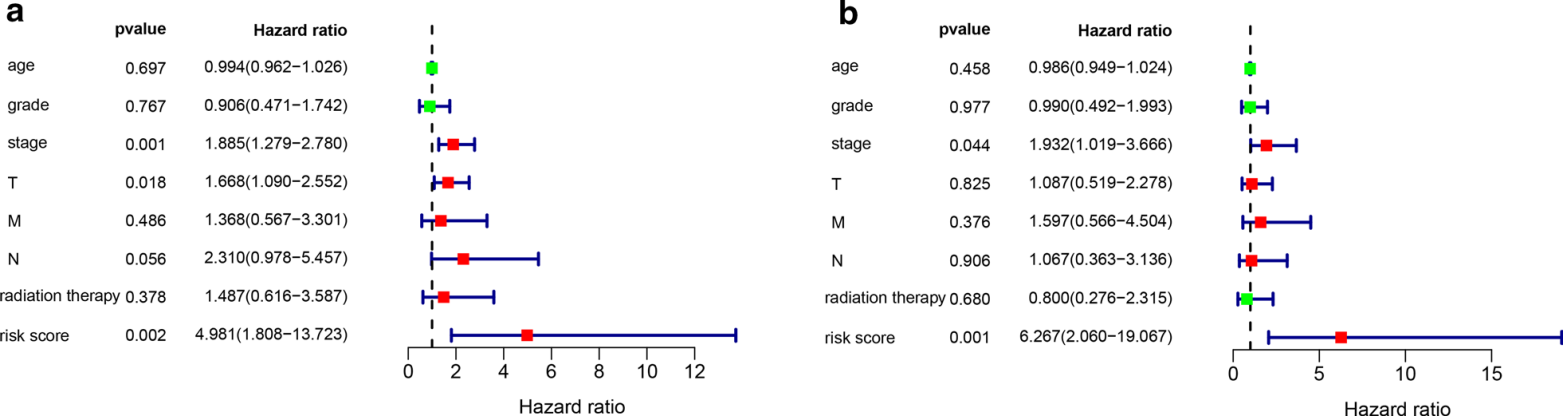
Univariate and multivariate Cox analyses of prognosis. **a** Univariate analysis. **b** Multivariate analysis

**Fig. 9:**
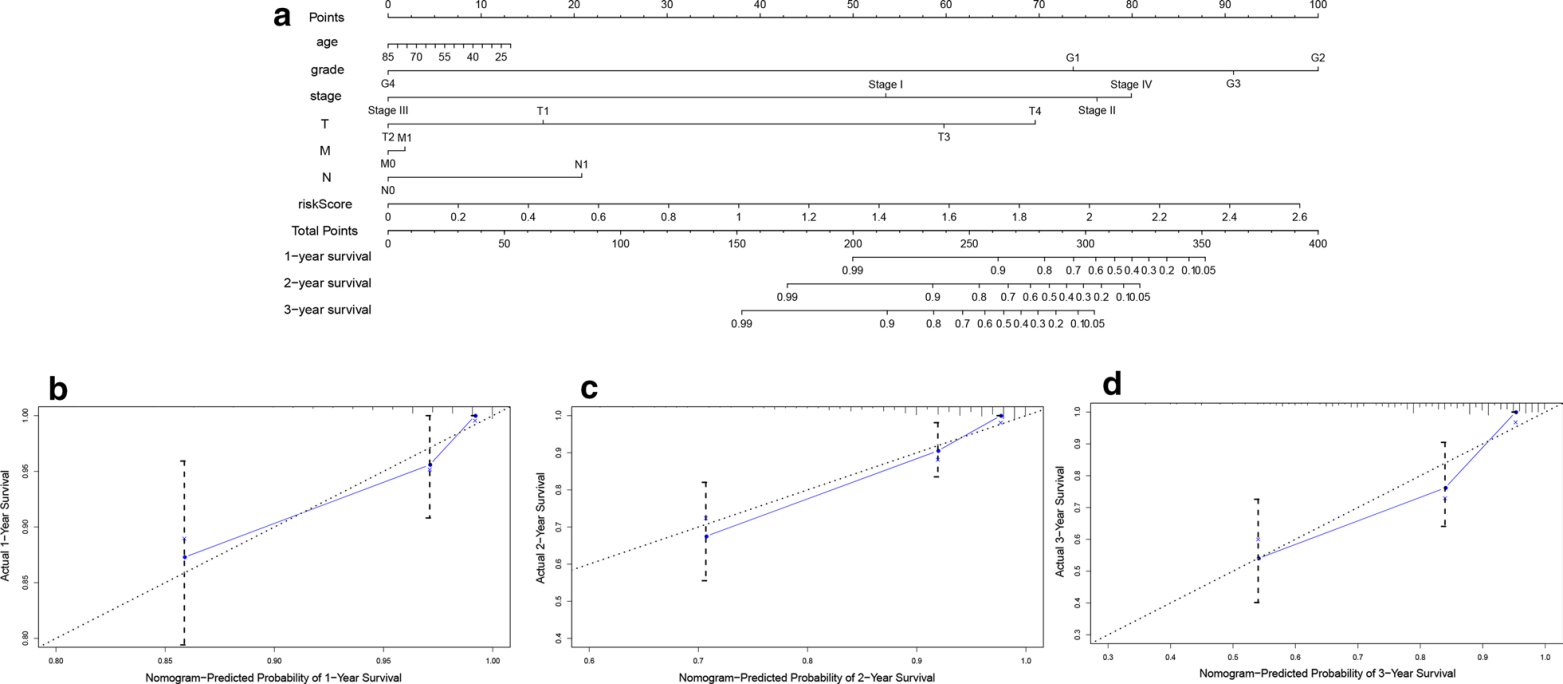
Nomogram and calibration plots predict prognosis in patients with cervical cancer. **a** The nomogram predicts prognosis based on the risk score and other clinical features. Calibration plot for (**b**) 1-year survival; **c** 2-year survival; **d** 3-year survival

Univariate analysis showed that TNM stage, T stage and risk score were associated with prognosis in patients with cervical cancer (Fig. 8a). After adjusting for age, grade, TNM stage, T stage, M stage, N stage, and radiation therapy of cervical cancer patients, multivariate analysis showed that the risk score remained an independent prognostic risk factor (Fig. 8b, HR = 6.267, 95% CI = 2.060–19.067, P = 0.001). Finally, we constructed a nomogram based on clinical features and risk score to assess the prognosis of cervical cancer patients (Fig. 9a), whereby a high score indicates that the patient has a poor prognosis. Calibration plots showed that the nomogram resulted in an accurate prognosis for patients with cervical cancer (Fig. 9b–d).

### Relationship between the three-gene signature and clinicopathological features

**Fig. 10:**
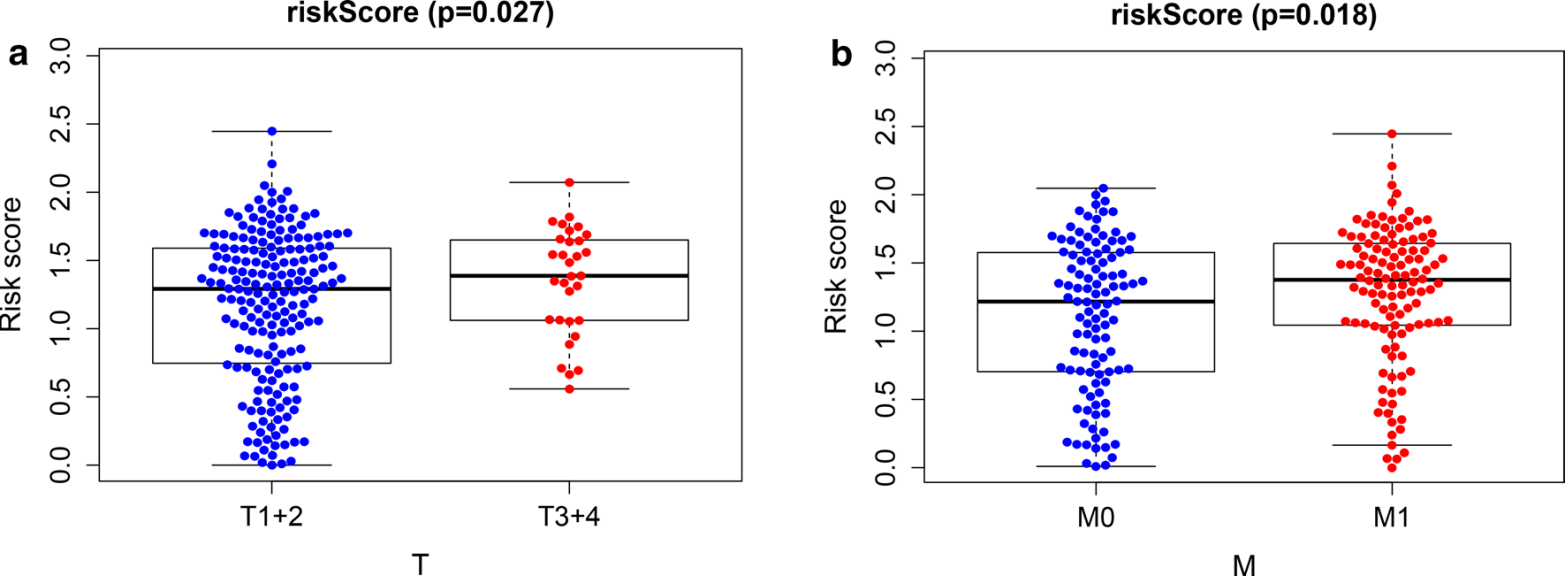
The relationship between risk score and (**a**) T stage and (**b**) M stage

**Fig. 11:**
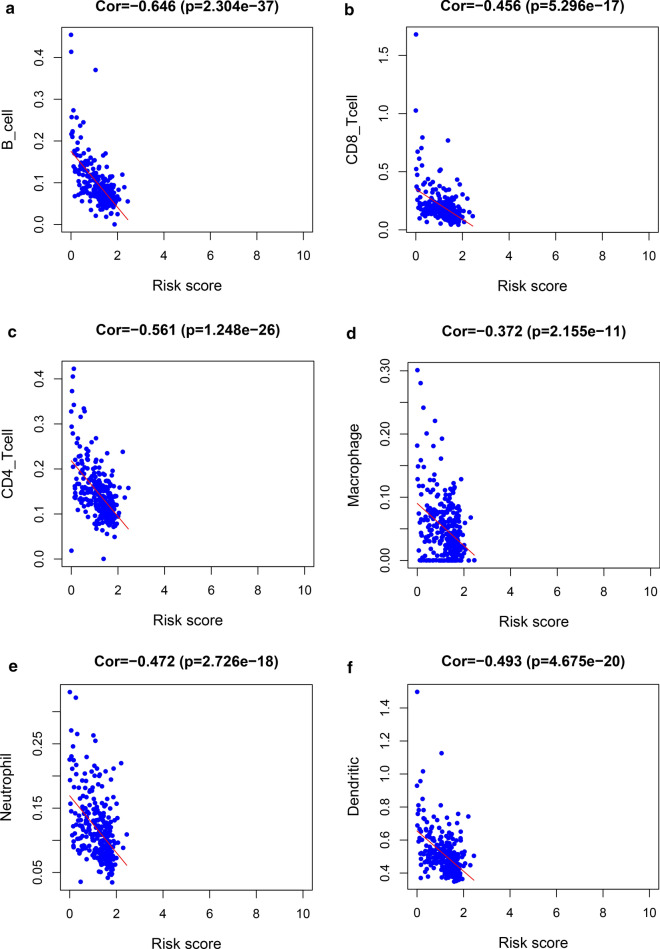
The relationship between risk score and immune infiltration. **a** B cells; **b** CD8 + T cells; **c** CD4 + T cells; **d** macrophages; **e** neutrophils; **f** dendritic cells

We analyzed the risk scores and clinical features of patients with cervical cancer, including age, grade, TNM stage, T stage, M stage, and N stage. The results showed a higher risk score in the high-grade T stage (P = 0.027) and metastatic tissues (P = 0.018) (Fig. 10). We also examined the relationship between risk score and immune cell infiltration levels and found that the risk score was significantly negatively correlated with the degree of infiltration of B cells, CD8 + T cells, CD4 + T cells, macrophages, neutrophils, and dendritic cells in CESC (Fig. 11).

## Discussion

CESC is one of the main causes of cancer death in females, especially in sub-Saharan Africa and Southeast Asia, with a mortality rate of more than 50%, which is 18 times that of developed countries [1, 3]. Because of the poor prognosis of cervical cancer, it is important to find molecular markers to predict prognosis. With the development of bioinformatics technology, an increasing number of researchers are evaluating the significance of multigene signatures for the prognosis of different tumors through open databases. For example, Yang et al. found that a seven-gene model can accurately predict the prognosis of head and neck squamous cell carcinoma [25]. Using lung cancer data from TCGA, a seven-gene model was able to predict prognosis [26]. An eight-gene prognostic model was reported to have predictive power for cervical cancer prognosis [19]. Ding et al. identified a potential three-gene signature (MSRB3, CENPM, ZIC2) that can predict the prognosis of cervical cancer through the TCGA dataset, which is expected to provide a basis for the prognostic evaluation of patients in the future [27]. The tumor microenvironment affects the prognosis and treatment of tumors, especially with regard to immunotherapy, though a multigene model associated with the tumor microenvironment (TME) has not been reported for cervical cancer prognosis.

In this study, we downloaded mRNA expression data and clinical follow-up information for 307 patients with cervical cancer from TCGA. We divided the cervical cancer samples into high and low immunity and matrix scores using the ESTIMATE algorithm and obtained 420 differential genes in the cervical cancer microenvironment. GO and KEGG analyses revealed differential gene enrichment in immune-related functions and pathways. In addition, we constructed a three-gene signature (SLAMF1, CD27, SELL) to predict prognosis based on Cox analysis in the training group. The overall survival time of the high-risk score group was shorter than that of the low-risk score group, including patients in different subgroups (age and stage). The three-gene signature was able to accurately predict the prognosis of cervical cancer. In addition, the results were further verified in the testing and combined groups. Multivariate Cox analysis showed that the three-gene signature was an independent risk factor for cervical cancer prognosis. Based on age, grade, TNM stage, T stage, M stage, N stage and risk score, a nomogram was constructed to score prognosis, and calibration plots showed that the nomogram could accurately predict patient survival. Furthermore, the results showed that the three-gene signature scored high for the high-grade T stage and metastatic tissue and was associated with the degree of immune infiltration in the TME.

SLAMF1, also known as CD150, belongs to the SLAM receptor family. SLAMF1 is commonly expressed in the immune system, and it was recently found to be expressed in tumors of the central nervous system [28]. A previous study showed that a two-gene prognostic model can accurately predict the prognosis of patients with chronic lymphocytic leukemia, in which SLAMF1 is a risk gene, and patients with high risk scores had a poorer prognosis than did those with low risk scores [29]. In breast cancer, Lin et al. suggested associations between allelic expression imbalance polymorphisms and breast cancer risk (SLAMF1 rs1061217) [30]. CD27, a member of the tumor necrosis factor receptor family, is present in CD4, CD8 T lymphocytes and NK cells, plays a significant role in tumor immunotherapy [31, 32]. In multiple myeloma, Kaplan–Meier analysis showed that patients with high CD27 expression had a longer overall survival time than patients with low CD27 expression [33]. SELL, also called CD62L, is located on the surface of T cells and promotes T cell homing to lymph nodes. Vlad et al. revealed that CD62L is downregulated in chronic lymphocytic leukemia and associated with disease progression [34]. Additionally, CD62L can enhance the efficacy of tumor immunotherapy for cancer treatment [35]. In bladder cancer, the serum level of SELL has been found to be useful as a tool for diagnostic staging and grading [36]. The results of these studies were consistent with our findings in CESC.

To investigate the mechanisms in the CESC microenvironment, we performed GO and KEGG analysis for the differentially expressed genes, as well as PPI network analyses and immune infiltration correlation analysis. The results revealed that genes were concentrated on immune-related pathways, such as the T cell receptor signaling pathway, the intestinal immune network for IgA production, and the B cell receptor signaling pathway. T cell receptor signaling has been reported to play a role in the progression of extranodal NK/T cell lymphoma and to activate the ITK/NF-κB/GATA-3 axis to promote chemoresistance in T cell lymphomas [37, 38]. Yang et al. found that the intestinal immune network for IgA production affects the cell proliferation and migration of liver cancer cells [39]. Krysiak et al. found that the B cell receptor signaling pathway affects the biology, prognosis, and treatment of follicular lymphoma [40]. Another study revealed that B cell receptor signaling affects the prognosis of patients with chronic lymphocytic leukemia [41]. Based on these results, the hub genes are speculated to play an important role in the CESC microenvironment.

However, there are still some limitations in our research. Although the prognostic significance of the three-gene signature in patients with cervical cancer was analyzed by bioinformatics, additional samples and external datasets will need to be further studied and verified. In our future work, we will externally verify the prognostic accuracy of the three-gene signature in other databases and local patient data via PCR. Functional testing is needed, and determining the mechanisms of the TME requires further research. Our research team is currently working towards some of these goals.

## Conclusions

For the first time, we analyzed CESC sample data using the ESTIMATE algorithm and found that a three-gene signature consisting of core genes in the cervical cancer microenvironment can be used to predict patient prognosis. In addition, the three-gene signature is an independent risk factor for the prognosis of cervical cancer. Gene functional analysis revealed associations with immune responses. Indeed, this research indicates that three-gene signature may be applied as biomarkers to predict the prognosis and therapeutic targets of CESC.

## Data Availability

Research Data are available at TCGA.

## Abbreviations

CESC: Cervical cancer
HR-HPV: High-risk human papillomavirus
TCGA: The cancer genome atlas
TIMER: Tumor immune estimation resource
ROC: Receiver operating characteristic
AUC: Area under the curve
TME: Tumor microenvironment
GO: Gene ontology
KEGG: Kyoto encyclopedia of genes and genomes
PPIs: Protein–protein interactions
MCODE: Molecular complex detection
BP: Biological process
CC: Cellular component
MF: Molecular function
log2FC: Log2-fold change
FDR: False discovery rate
